# Genome-Resolved Delineation of Three Novel Endophytic *Achromobacter* Species from Desert Medicinal Plants

**DOI:** 10.3390/microorganisms14051019

**Published:** 2026-04-30

**Authors:** Khadija Ait Si Mhand, Salma Mouhib, Juan Carlos Fernández-Cadena, Mohamed Hijri

**Affiliations:** 1 African Genome Center, University Mohammed VI Polytechnic (UM6P), Lot 660, Hay Moulay Rachid, Ben Guerir 43150, Morocco; khadija.aitsimhand@um6p.ma (K.A.S.M.); salma.mouhib@um6p.ma (S.M.); 2 OMICS Sciences Laboratory, Faculty of Health Science, Universidad Espíritu Santo, Samborondón 092301, Ecuador; jfernandezcadena@bwh.harvard.edu; 3 Harvard Medical School and Brigham and Women’s Hospital, Boston, MA 02115, USA; 4 Institut de Recherche en Biologie Végétale (IRBV), Département de Sciences Biologiques, Université de Montréal, 4101 Rue Sherbrooke Est, Montréal, QC H1X 2B2, Canada

**Keywords:** endophytic bacteria, arid environment, sustainable agriculture, whole-genome sequencing, novel bacterial species

## Abstract

Endophytic bacteria from plants adapted to arid and semi-arid environments represent an underexplored reservoir of microbial diversity with potential agricultural applications. Here, we report a polyphasic taxonomic and genome-based characterization of *Achromobacter* sp. isolates recovered from root and foliar tissues of *Citrullus colocynthis* and *Peganum harmala*, two medicinal plants thriving under harsh environmental conditions. Whole-genome sequencing, phylogenomic analyses, average nucleotide identity (ANI), digital DNA–DNA hybridization (dDDH), multilocus sequence typing, and detailed phenotypic profiling revealed three previously undescribed species, for which we propose the names *Achromobacter colocynthi* sp. nov., *Achromobacter maghribensis* sp. nov., and *Achromobacter semiaridum* sp. nov. Genome assemblies were highly complete (98.7–99.2%) with minimal contamination (<1%), supporting robust taxonomic inference. All three species displayed ANI and dDDH values below accepted thresholds relative to their closest phylogenetic neighbors, despite partial inconsistencies in 16S rRNA similarity for one isolate, highlighting the value of genome-wide metrics for species delineation. Phylogenomic analyses placed the novel taxa within *Achromobacter* sp. as distinct evolutionary lineages. Phenotypic characterization indicated broad metabolic versatility, including utilization of carbohydrates, organic acids, and amino acids, tolerance to moderate salinity and acidic pH, and resistance to multiple antimicrobial compounds, traits likely linked to adaptation to endophytic lifestyles under semi-arid conditions. Beyond their taxonomic novelty, the isolates exhibited in vitro traits associated with plant adaptation and stress tolerance, including IAA production, ACC deaminase activity, and tolerance to Zn, Cu, and Cd. Genomic analyses further indicated functions related to phosphate acquisition and stress response. These findings expand the taxonomic framework of *Achromobacter* sp., establish *C. colocynthis* and *P. harmala* as reservoirs of novel endophytic bacteria, and highlight their potential relevance for agricultural biotechnology in stress-prone environments.

## 1. Introduction

Plant-associated microbiomes play a central role in plant nutrition, stress resilience, and ecosystem functioning, making them key targets for sustainable agricultural and environmental biotechnology [1,2]. Among the diverse bacterial taxa interacting with plants, members of the genus *Achromobacter* (family Alcaligenaceae) are increasingly recognized as versatile and functionally important plant-associated bacteria. These Gram-negative bacteria inhabit a wide range of soil environments, including extreme and disturbed ecosystems characterized by high salinity, alkalinity, drought, or heavy metal contamination, and colonize plants as rhizosphere inhabitants, endophytes, or phyllosphere residents [3,4,5].

Accumulating evidence demonstrates that *Achromobacter* species function as effective plant-growth-promoting rhizobacteria (PGPR), contributing to plant productivity through multiple, often complementary, mechanisms [6,7]. A hallmark feature of several *Achromobacter* sp. strains is their ability to modulate plant hormone signaling, particularly auxin dynamics. Certain isolates alter auxin distribution within root tissues, reshaping root architecture by enhancing primary root elongation and lateral root formation, even under abiotic stress conditions such as salinity or alkalinity [5,8,9]. In addition, many strains produce indole-3-acetic acid (IAA) or regulate plant hormone homeostasis through the catabolism of excess auxins or ethylene precursors via 1-aminocyclopropane-1-carboxylate (ACC) deaminase activity, thereby alleviating stress-induced growth inhibition [4,10,11].

Beyond hormone modulation, *Achromobacter* sp. enhance nutrient acquisition through phosphate solubilization, siderophore-mediated iron uptake, and, in some cases, nitrogen metabolism, improving plant nutritional status in nutrient-limited soils [3,10,12]. These traits are particularly valuable in low-input or degraded agroecosystems, where nutrient availability constrains crop productivity. Moreover, the ability of *Achromobacter* sp. strains to mitigate abiotic stress, through modulation of ethylene signaling, enhancement of antioxidant enzyme activities, and regulation of ion homeostasis, confers increased tolerance to drought, salinity, and trace element exposure [5,11,13].

In addition to direct growth promotion, several *Achromobacter* sp. strains exhibit strong biocontrol potential against soil-borne pathogens [14]. Endophytic and rhizosphere-associated isolates have been shown to suppress fungal pathogens such as Rhizoctonia solani and *Fusarium oxysporum* through the production of antifungal metabolites or lytic enzymes or by inducing systemic resistance responses in host plants [15,16]. These disease-suppressive properties position *Achromobacter* sp. as promising candidates for integrated plant health management strategies.

The ecological versatility of *Achromobacter* sp. extends beyond agriculture into environmental biotechnology. Numerous strains isolated from contaminated soils possess the capacity to immobilize, transform, or detoxify trace elements such as copper, cadmium, and chromium, while simultaneously supporting plant growth, an attribute that is particularly valuable for phytoremediation applications [10,11]. Furthermore, *Achromobacter* sp. are capable of degrading a wide range of organic pollutants, including pesticides, herbicides, and polycyclic aromatic hydrocarbons, thereby contributing to soil detoxification and ecosystem restoration [17,18,19].

From a community ecology perspective, *Achromobacter* sp. is frequently detected as a core or keystone taxon across diverse rhizosphere and soil microbiomes, influencing microbial community structure and function [20,21]. These bacteria often operate within synergistic microbial consortia, where interactions with other beneficial microbes enhance disease suppression, nutrient cycling, or pollutant degradation, suggesting that consortium-based applications may outperform single-strain inoculants under field conditions [22,23].

Recent advances in genome sequencing have provided deeper insights into the genetic basis underlying the ecological success and functional versatility of *Achromobacter* sp. Comparative genomic analyses reveal a rich repertoire of genes involved in hormone synthesis and catabolism, phosphate solubilization, siderophore production, stress tolerance, antimicrobial resistance, and xenobiotic degradation, explaining their adaptability across diverse environmental niches [4,5]. Genome-based taxonomy has also revealed substantial hidden diversity within the genus, indicating that many environmentally and agriculturally relevant *Achromobacter* sp. lineages remain undescribed.

Despite growing interest, important knowledge gaps remain. Field-scale efficacy of *Achromobacter*-based inoculants is variable and context-dependent, influenced by host plant identity, soil conditions, and competition with native microbiota. Long-term ecological impacts, persistence, and biosafety considerations also require careful evaluation prior to widespread agricultural deployment. Moreover, the taxonomic and functional diversity of *Achromobacter* sp. endophytes associated with stress-adapted plants from arid and semi-arid ecosystems remains poorly explored.

*Citrullus colocynthis* (L.) Schrader (Cucurbitaceae) and *Peganum harmala* L. are two desert-adapted medicinal and wild plants distributed across arid and semi-arid regions of North Africa, the Middle East, and parts of Asia, where they persist under drought, salinity, and nutrient-limited conditions. They represent a particularly rich reservoir of stress-tolerant endophytic bacteria shaped by chronic water limitation, high salinity, nutrient scarcity, and thermal stress. Recent genome-based studies have highlighted the importance of this plant as a niche for previously undescribed microbial lineages with specialized adaptations to dryland ecosystems. Notably, references [24,25] reported two novel endophytic *Microbacterium* species isolated from *C. colocynthis* and one *Acenitobacter* species isolated from *P. harmala* in arid Morocco, revealing genomic traits associated with endophytism under xeric conditions, including oxidative and osmotic stress responses, metal homeostasis, phosphate acquisition, polyphosphate metabolism, and siderophore uptake. These studies further demonstrated ecological differentiation among endophytes, with contrasts in nitrogen metabolism and carbon utilization strategies suggesting niche partitioning within the host plant. Together, these findings underscore *C. colocynthis* and *P. harmala* as a reservoir of indigenous, functionally specialized endophytes and emphasize the value of genome-resolved taxonomy for uncovering microbial diversity in underexplored arid regions.

The present study employed a polyphasic and genome-resolved approach to characterize *Achromobacter* sp. strains isolated from *C. colocynthis* and *P. harmala*. By integrating whole-genome sequencing, phylogenomic analyses, and detailed phenotypic characterization, we describe three novel *Achromobacter* species and provide insights into their ecological adaptation and functional potential in dryland plant-associated niches. By expanding taxonomic coverage beyond *Actinobacteria* to include Proteobacteria-associated endophytes, our findings broaden current understanding of *Achromobacter* sp. diversity and reinforce the concept that arid-adapted plants harbor unique microbial resources with relevance for agricultural and environmental biotechnology. Collectively, these results contribute region-specific genomic references from North African semi-arid ecosystems and highlight the potential of indigenous endophytes as bioregion-appropriate bioinoculants for enhancing crop resilience and soil health under increasing environmental stress.

## 2. Materials and Methods

### 2.1. Sampling and Bacterial Isolation

Endophytic *Achromobacter* sp. strains were isolated from root and leaf tissues of *Citrullus colocynthis* collected at flowering from a site along a decommissioned wastewater canal near Green City, Benguerir, Morocco (32°11′48.6″ N, 7°56′30.0″ W), and from *Peganum harmala* sampled from a natural population in Nzalat Laadam, Benguerir, Morocco (32°06′49.6″ N, 7°57′13.0″ W). Sampling was conducted on 27 May 2022 and 23 July 2023. Plant material was transported to the laboratory under cooled conditions and processed immediately.

Surface sterilization and isolation followed previously described workflows with minor adaptations [24,25,26]. Plant tissues were surface sterilized using a three-step procedure consisting of immersion in 2% (*v*/*v*) Tween 20 for 3 min, followed by rinsing in sterile distilled water for 1 min, then immersion in 2% sodium hypochlorite for 3 min, and finally three successive washes with sterile distilled water (1 min each). Sterilized tissues were air-dried inside an aseptic flow cabinet and cut into fragments of approximately 5 mm^2^ using a sterile scalpel. The effectiveness of surface sterilization was verified by tissue-imprint controls and by plating the final rinsing water onto the same agar media used for isolation. The absence of microbial growth in these controls confirmed successful surface disinfection and supported the endophytic origin of the isolates. Sterilized tissue fragments were plated onto Tryptic Soy Agar (TSA) and Potato Dextrose Agar (PDA), both at full strength (1×) and one tenth strength (1/10×). In addition, one set of fragments was overlaid with sugar free minimal (M) medium to broaden recovery. Plates were incubated at 28 °C for up to one month, and emerging colonies with distinct morphologies were periodically subcultured until pure. Purified isolates were preserved in Tryptic Soy Broth (TSB) medium supplemented with 25% glycerol at −80 °C for long term storage.

### 2.2. DNA Extraction, 16S rDNA Sequencing and Whole-Genome Sequencing

Genomic DNA was extracted from fresh cultures using a standardized protocol described previously [25]. Preliminary identification was performed by PCR amplification and Sanger sequencing of the 16S rRNA gene [24]. For whole-genome sequencing, DNA from three representative isolates was used to prepare Nextera XT libraries (Illumina, San Diego, CA, USA) with 200 ng input DNA and unique dual indices. Libraries were sequenced in a 2 × 151 bp paired-end configuration. Reads were demultiplexed using bcl2fastq v2.17.1.14 prior to downstream analyses.

### 2.3. Genome Assembly, Annotation, Taxonomic Analyses, and Functional Profiling

The bioinformatics analysis workflow was described in detail by Mouhib, et al. [25] and Ait Si Mhand, et al. [24]. Raw reads were quality-filtered and trimmed using BBDuk74 with a minimum Phred quality score of 20 and a minimum read length of 100 bp and were assembled de novo using MaSuRCA v4.1.4. Assembly quality was evaluated using QUAST v5.3.0, and genome completeness and contamination were assessed using CheckM v1.2.0. The highest quality draft genome for each strain was retained for downstream analyses.

Taxonomic assignment combined 16S rRNA gene comparisons with genome-based relatedness indices against the closest type strains, complemented by ribosomal protein MLST profiling. Genomes were annotated using Prokka v1.14.6, core-genome analysis was conducted with Roary v 3.13.0, and phylogenomic relationships were inferred from concatenated single-copy core genes. A maximum-likelihood tree was reconstructed with IQ-TREE 2 using ModelFinder and 1000 ultrafast bootstrap and 1000 SH-aLRT replicates. Partial 16S rRNA gene phylogeny was inferred using MAFFT v 7.526 alignment and FastTree v 2.2.0 under the GTR + Gamma model. Trees were visualized in iTOL v 7.

Genome-based species delineation was interpreted using the widely accepted thresholds of approximately 95–96% ANI and 70% dDDH. The ANI and dDDH values obtained for the strains analyzed here were below these accepted species-level cutoffs relative to their closest validly described type strains, supporting their recognition as novel species. Genome annotation was further complemented by KEGG Orthology assignment using KofamKOALA v 115.0. A curated list of genes associated with endophytic and plant-growth-promoting functions was used to identify candidate loci, and gene counts were normalized as z-scores and visualized in R v 4.6.0.

### 2.4. Whole-Genome Comparison and Visualization

Genome visualization and comparative circular maps were generated using Proksee v1.14.6 [27], followed by KEGG Orthology assignment using KofamKOALA v 115.0 [28]. GC content, GC skew, mobile genetic elements (MobileOG-db v 1.1.3) [29], and antimicrobial resistance genes (CARD) were annotated. Draft genomes were compared to their closest type strains using BLASTn v 2.17.0, with similarity displayed as percent identity bins (≥85%, ≥90%, ≥95%, 100%). These analyses provided qualitative assessment of genome-wide similarity and localized divergence.

### 2.5. Phylogenetic and Phylogenomic Analyses

Partial 16S rRNA gene sequences were aligned using MAFFT [30], and maximum-likelihood trees were inferred with FastTree 2.2.0 (GTR + Gamma) [31]. Trees were visualized in iTOL v7 [32].

For phylogenomics, genomes were annotated with Prokka v1.14.6 and analyzed using Roary to define the core genome [33]. A concatenated nucleotide alignment was used for maximum-likelihood inference in IQ-TREE 2 2.4.0 with ModelFinder. Node support was assessed using 1000 ultrafast bootstrap and 1000 SH-aLRT replicates [34].

All analyses were performed in a Linux HPC environment using consistent software versions and parameters.

### 2.6. Phenotypic Characterization

Phenotypic profiling was conducted using the GEN III MicroPlate system (Biolog, Hayward, CA, USA), assessing carbon utilization and chemical sensitivity. Metabolic activity (A590) was recorded every 24 h for up to 7 days and compared with reference database profiles [35].

### 2.7. Screening of Plant-Growth-Promoting Traits

PGP traits were assessed using qualitative and semi-quantitative assays. Indole-3-acetic acid (IAA) production was measured using Salkowski reagent [36]. ACC deaminase activity was screened via α-ketobutyrate detection [37]. Phosphate solubilization was evaluated on Pikovskaya agar [38]. Iron acquisition was assessed using CAS agar and M9 medium supplemented with 2,2′-dipyridyl [39,40].

Metal tolerance (Cu, Cd, Zn; 0–20 mM) was tested in liquid culture with growth monitored at OD600. Hydrogen cyanide production was evaluated using a picrate-based assay. Results were recorded as positive (+), weak (W), or negative (−) [41].

### 2.8. Statistical Analysis

Quantitative screening data for plant-growth-promoting traits are presented as mean values based on replicate measurements. As these assays were intended for screening purposes within the context of species characterization, the results were interpreted descriptively rather than subjected to formal inferential statistical analyses.

## 3. Results

### 3.1. Isolation and Growth Characteristics of Endophytic Achromobacter sp.

A culture-dependent approach was employed to isolate putative endophytic bacteria from root and leaf tissues of *Citrullus colocynthis* and *Peganum harmala*. Sanger sequencing of the 16S rRNA gene from the purified isolates identified ten strains belonging to the genus *Achromobacter* sp. All strains were selected for comprehensive characterization because they did not exhibit sufficient sequence identity to any previously described species, with the exception of strain AGC27, which showed 98.4% sequence similarity to *Achromobacter piechaudii* but low sequence coverage (94.33%; Table S1).

Eight isolates originated from *C. colocynthis*, whereas two were recovered from *P. harmala*. Among the *C. colocynthis* isolates, seven strains (AGC39, AGC93, AGC45, AGC61, AGC86, AGC78, and AGC69) were obtained from roots and one strain (AGC14) from leaves. From *P. harmala*, one strain (AGC27) was isolated from roots and one (AGC25) from leaves. Under the tested culture conditions, all isolates displayed similar colony morphology and comparable baseline growth characteristics.

16S rRNA gene analysis classified nine isolates as *Achromobacter* sp. (Table S1), with 99–100% sequence coverage and 96.86–100% identity. A phylogenetic reconstruction based on the 16S rRNA gene (Figure S1) supported their placement at the genus level but provided limited resolution at the species level. To refine taxonomic classification and determine whether these isolates represent novel species, whole-genome sequencing and genome-based analyses were subsequently performed.

### 3.2. Genome Characteristics of the Novel Achromobacter sp. Isolates

Draft genome assemblies of the three proposed type strains (AGC39. AGC45 and AGC69) ranged from 6.32 to 6.67 Mb, with GC contents between 64.20% and 64.97% (Tables S2 and S3). Genome completeness was high (98.74–99.07%) except for one isolate with low contamination levels (0.53–1.32%).

Non-type strains assigned to each proposed species exhibited comparable genomic features. For isolates AGC14 and AGC93, assigned to the candidate *Achromobacter semiaridum* type strain AGC39, genome sizes ranged from 6.19 to 6.32 Mb, with GC contents of 64.71–64.85% and completeness > 98%. Isolates AGC61, AGC78, and AGC86, assigned to the candidate *Achromobacter colocynthi* type strain AGC45, had genome sizes between 6.38 and 6.70 Mb, GC contents of 64.17–64.24%, and completeness values of 98.83–99.58%. Isolates AGC25 and AGC27, assigned to the candidate *Achromobacter maghribensis* type strain AGC69, had genome sizes ranging from 6.22 to 6.32 Mb, GC contents of 64.16–64.97%, and completeness values of 81.48–99.02% (Tables S2 and S3).

**Figure 1:**
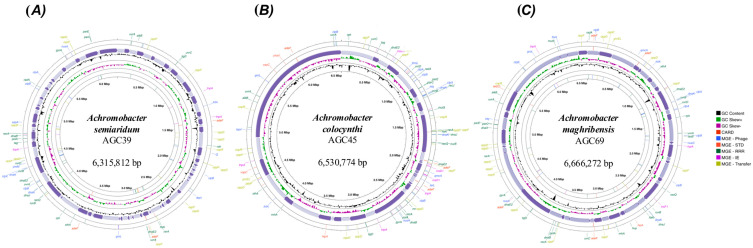
Genome maps of *Achromobacter* sp. nov. type strains. The outermost rings indicate genome annotations for mobile genetic elements (MGEs), categorized into phage, Stability–Transfer–Defense (STD), Replication–Recombination–Repair (RRR), Integration–Excision (IE), and transfer functions. Purple arrows represent the contigs assembled for each strain. GC content and GC skew (+/−) are shown in the inner tracks. The innermost circle highlights antimicrobial resistance genes annotated using the CARD database. The inner coordinate ring indicates genome position in megabases (Mbp), and the strain name and total assembly size are shown at the center of each map.

Circular genome maps of type strains AGC39, AGC45, and AGC69 illustrate overall genome organization, GC content and GC skew profiles, and the distribution of annotated mobile genetic-element-associated regions (Figure 1). All three genomes exhibit largely continuous chromosomal backbones with coding sequences distributed throughout, although strain-specific mobile-element–associated regions were detected at distinct chromosomal locations. Predicted coding sequences, tRNA counts, rRNA gene recovery, and additional assembly statistics for all isolates are summarized in Table S3.

### 3.3. Genome-Based Taxonomy and Phylogenomics

Multilocus sequence typing (MLST) and genome relatedness indices supported the distinction of the isolates from previously described *Achromobacter* species. The ten isolates analyzed in this study resolved into three species-level groups based on concordant genome-based evidence, including ANI, dDDH, and phylogenomic clustering. After conspecificity within each group was established, one representative strain per group was selected for detailed downstream characterization. In each case, the proposed type strain was chosen based on superior genome assembly quality, including more favorable contiguity metrics and N50 values.

Strains AGC39, AGC93, and AGC14 formed a coherent cluster representing *Achromobacter semiaridum* sp. nov. Their closest relative was *Achromobacter piechaudii*, with an ANI of 95.50% and dDDH of 57% for the type strain AGC39 (Table 1). Although ANI approached the species threshold, dDDH values were well below the 70% cutoff, supporting species-level separation.

Strains AGC45, AGC61, AGC86, and AGC78 were assigned to *Achromobacter colocynthi* sp. nov., most closely related to *Achromobacter spanius* (ANI 94.00–94.40%; dDDH ~50%).

Strain AGC69 was classified as *Achromobacter maghribensis* sp. nov., with *Achromobacter marplatensis* as its nearest relative (ANI 91.00%; dDDH 39%). Additionally, two independent isolates assigned to the same proposed lineage as *A. maghribensis* were recovered from both root and leaf tissues of *P. harmala*. Genome comparison against *A. marplatensis* yielded ANI values of 91% and dDDH values of 38.8–38.9% (MLST similarity: 64%) (Table S2), supporting their inclusion within the *A. maghribensis* species framework and extending its host range.

**Figure 2:**
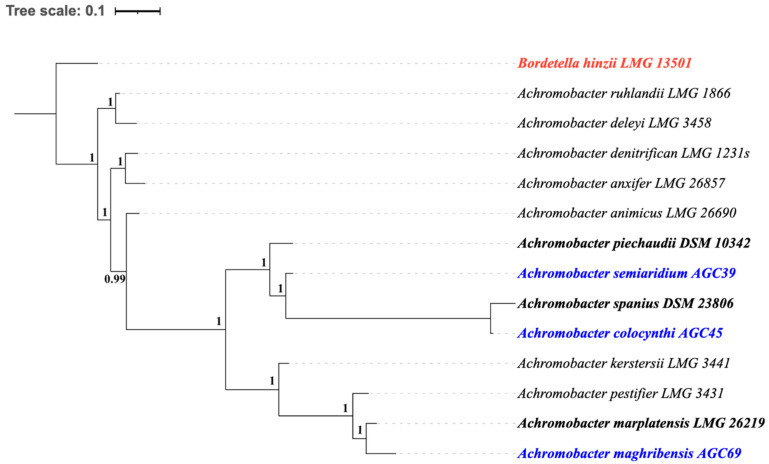
Genome-based phylogenomic tree showing the relationships between the novel *Achromobacter* strains AGC39, AGC45, and AGC69 and closely related, validly published *Achromobacter* species. The tree was inferred using a maximum-likelihood approach based on conserved single-copy core genes and is midpoint-rooted. Branch support values are indicated at the nodes as normalized values, where 1 corresponds to 100% support. Novel strains are highlighted in blue, the outgroup is in red, and the closest relative in black Bold. The scale bar represents substitutions per site.

Core-genome phylogenomic analysis based on whole-genome alignments resolved the three type strains (AGC39, AGC45, AGC69) into three well-supported and distinct lineages within *Achromobacter* sp., clearly separated from their closest type strains (Figure 2).

### 3.4. Integrated Phenotypic and Genomic Characterization

**Figure 3:**
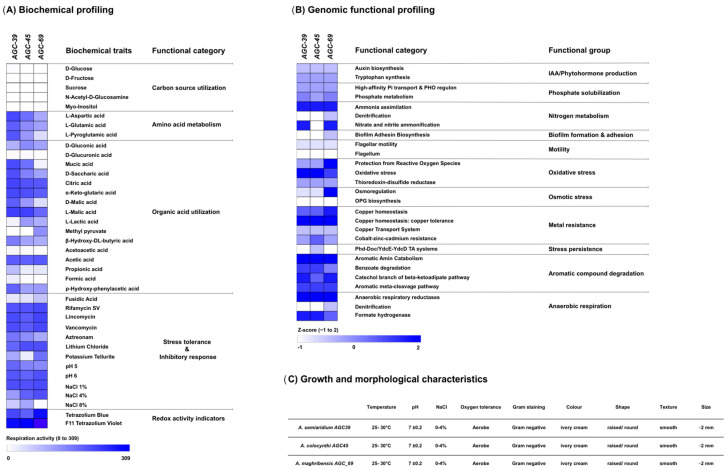
Integrated biochemical, genomic functional, and growth-related characterization of the novel *Achromobacter* type strains AGC39, AGC45, and AGC69. (**A**) Biochemical profiling based on substrate utilization patterns determined using Biolog assays. (**B**) Genomic functional profiling derived from genome annotation, highlighting major functional categories. (**C**) Growth performance and morphological characteristics of the novel strains.

Phenotypic traits, biochemical responses, and genome-derived functional potential were jointly assessed for the three proposed type strains (*A. semiaridum* AGC39, *A. colocynthi* AGC45, and *A. maghribensis* AGC69) (Figure 3). Biochemical profiling using Biolog GEN III plates revealed broadly similar metabolic activity patterns across the three strains, with respiration observed across multiple substrate classes and chemical sensitivity conditions (Figure 3A). While many reactions were shared, strain-level variation was evident in the intensity of respiration signals for selected amino acids, organic acids, and several inhibitory-stress conditions, consistent with species-level phenotypic differentiation.

Genome-based functional profiling identified a conserved core of functional categories across the three type strains, alongside notable differences in several adaptive and metabolic traits (Figure 3B). All three genomes encoded functions related to tryptophan-associated and auxin-related metabolism, as well as phosphate acquisition, indicating a shared functional backbone.

In contrast, functions associated with nitrogen metabolism were unevenly distributed. While basic nitrogen assimilation capacities were present in all strains, additional nitrogen-related functions were detected in *A. colocynthi* AGC45 and *A. maghribensis* AGC69 but absent in *A. semiaridum* AGC39. Similarly, traits linked to biofilm formation and motility showed species-specific patterns, varying in presence and relative representation rather than forming a fully conserved set.

Stress-related functional categories were broadly represented but differed among strains. Genes associated with oxidative stress response, metal resistance, and aromatic compound degradation were present in all three genomes, whereas osmotic stress adaptation functions were reduced in *A. semiaridum* AGC39 and *A. colocynthi* AGC45 but maintained in *A. maghribensis* AGC69.

Basic growth and colony characteristics were consistent across the three type strains, with growth observed at 25–30 °C, near-neutral pH (7 ± 0.2), and tolerance to up to 4% NaCl under aerobic conditions (Figure 3C). All strains were Gram-negative and formed ivory-cream colonies that were smooth, circular, raised, and approximately 2 mm in diameter (Figure 3C).

Collectively, these integrated phenotypic and genomic data provide a concise overview of functional potential and support the differentiation of the three novel *Achromobacter* species.

### 3.5. Comparative Circular Genome Mapping Against Closest Type Strains

**Figure 4:**
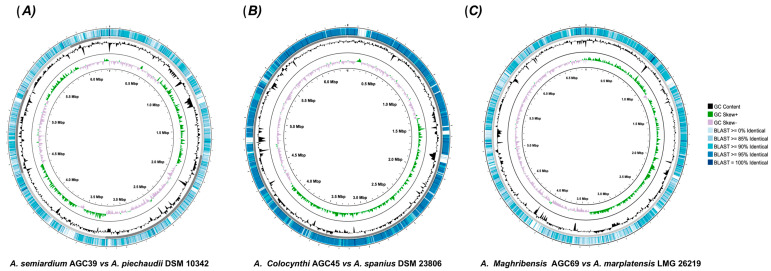
Comparative circular genome maps of the novel *Achromobacter* strains and their closest phylogenetic relatives. The maps display BLASTn similarity (outer ring; percent identity bins), GC content (black plot), GC skew (green/purple) of the reference genome and the inner coordinate ring indicating genome position in megabases (Mbp) used as the backbone. Regions of reduced similarity indicate localized genomic divergence and/or absence in the query genomes. (**A**) *Achromobacter semiaridium* AGC39 compared with *A. piechaudii* DSM 10342, (**B**) *Achromobacter colocynthi* AGC45 compared with *A. spanius* DSM 23806, and (**C**) *Achromobacter maghribensis* AGC69 compared with *A. marplatensis* LMG 26219.

Comparative circular genome maps illustrate BLASTn similarity of the novel *Achromobacter* sp. genomes against their closest type-strain reference backbones (Figure 4). Outer rings represent nucleotide identity classes (≥85%, ≥90%, ≥95%, and 100%), while inner rings display reference GC content and GC skew. AGC45 is enriched in high-identity regions, predominantly ≥95%, AGC69 shows mainly ≥90% alignments, and AGC39 exhibits a mixed profile with roughly equal representation of ≥85% and ≥90% identity classes. Overall, all three genomes display broad genome-wide synteny, with strain-specific differences in the distribution of identity classes.

### 3.6. Plant-Growth-Associated Trait Screening

The three type strains exhibited distinct but partially overlapping plant-growth-associated phenotypes (Table 2). ACC deaminase activity was consistently detected in all strains. In contrast, indole-3-acetic acid (IAA) production showed species-dependent variation, with no detectable activity in *A. semiaridum* AGC39, weak activity in *A. colocynthi* AGC45, and clear positive activity in *A. maghribensis* AGC69.

None of the strains demonstrated phosphate solubilization on Pikovskaya medium or growth under iron-chelating conditions (CAS blue and M9 + DIP). All three strains tolerated heavy metals, although with quantitative differences: copper tolerance was highest in AGC45 and AGC69 (up to 10 mM), whereas AGC39 tolerated copper up to 5 mM. Cadmium tolerance followed a similar pattern, with AGC45 and AGC69 tolerating up to 10 mM and AGC39 up to 5 mM, while zinc tolerance was uniformly high across all strains (5 mM).

Hydrogen cyanide (HCN) production was not detected in any of the three type strains. Collectively, these results indicate a conserved core of stress-related and ethylene-modulating traits, accompanied by strain-specific variation in auxin production and metal tolerance.

### 3.7. Species Protologues

#### 3.7.1. *Achromobacter colocynthi* sp. nov.

Etymology: *co.lo.cyn’thi*. M.L. neut. adj. *colocynthi*, referring to *Citrullus colocynthis*, the host plant from which the organism was isolated.

Type strain: AGC45^T^ (=CCMM B1338)

Type locality and source: Morocco; isolated from root tissue of *Citrullus colocynthis* growing in a semi-arid ecosystem.

Description: Cells are Gram-negative, aerobic, non-spore-forming rods. Colonies on Tryptic Soy Agar are smooth, circular, and cream-colored under the tested conditions (Figure 3C). Growth occurs at moderate temperatures under aerobic conditions, at near-neutral pH, with tolerance to moderate salinity. Biolog metabolic profiling is shown in Figure S2.

Genome features: Draft genome size ~6.2 Mb; DNA G + C content 64.71 mol%.

Accession IDs: 16S rRNA gene, PV706299; whole genome, SRR29855759.

BioProject/BioSample: PRJNA1133887; SAMN43406668

#### 3.7.2. *Achromobacter maghribensis* sp. nov.

Etymology: *mag.hri.ben’sis*. M.L. neut. adj. *maghribensis*, pertaining to the Maghreb region (North Africa), indicating the geographic origin.

Type strain: AGC69^T^ (=CCMM B1340)

Type locality and source: Morocco; isolated from root tissue of *Citrullus colocynthis* growing in a semi-arid ecosystem.

Description: Cells are Gram-negative, aerobic, non-spore-forming rods. Colonies on Tryptic Soy Agar are smooth, circular, and cream-colored under the conditions tested (Figure 3C). Growth occurs at moderate temperatures under aerobic conditions, at near-neutral pH, with tolerance to moderate salinity, Biolog results are shown in Figure S3.

Genome features: Draft genome size ~6.5 Mb; DNA G + C content 64.24 mol%.

Accession IDs: 16S rRNA gene, PV706313; whole genome, SRR29855774.

BioProject: PRJNA1133887; Biosample: SAMN43406623

#### 3.7.3. *Achromobacter semiaridum* sp. nov.

Etymology: *se.mi.a.ri’dum*. M.L. neut. adj. *semiaridum*, referring to the semi-arid environment from which the organism was isolated.

Type strain: AGC39^T^ (=CCMM B1336).

Type locality and source: Morocco; isolated primarily from root tissue of *Citrullus colocynthis* growing in a semi-arid ecosystem.

Description: Cells are Gram-negative, aerobic, non-spore-forming rods. Colonies on Tryptic Soy Agar are smooth, circular, and cream-colored under the conditions tested (Figure 3C). Growth occurs at moderate temperatures under aerobic conditions, at near-neutral pH, with tolerance to moderate salinity, Biolog results are shown in Figure S4.

Genome features: Draft genome size ~6.3 Mb; DNA G + C content 64.71 mol%.

Accession IDs: 16S rRNA gene, PV706298; whole genome, SRR29855769.

BioProject: PRJNA1133887; Biosample: SAMN43406634

## 4. Discussion

The combined evidence from genome-resolved taxonomy, comparative genomics, and phenotypic screening supports the recognition and characterization of three novel *Achromobacter* species, primarily recovered from root tissues of *Citrullus colocynthis* growing in a semi-arid region of Morocco. In contrast to many previously reported *Achromobacter* species described from clinical or other environmental sources, the strains characterized here were isolated as endophytes from arid-adapted host plants, highlighting an underexplored ecological context for diversification within the genus. Genome-scale analyses resolved these isolates into three previously undescribed species: *Achromobacter semiaridum* sp. nov. (AGC39, AGC93, and AGC14), *Achromobacter colocynthi* sp. nov. (AGC45, AGC61, AGC86, and AGC78), and *Achromobacter maghribensis* sp. nov. (AGC69). Their genomes range in size from approximately 6.19 to 6.70 Mb and exhibit GC contents and coding sequence numbers within the range reported for the genus *Achromobacter* sp. (NCBI Datasets, accessed 3 February 2026).

Notably, support for *A. maghribensis* sp. nov. extends beyond *C. colocynthis*, with two additional isolates recovered from wild *Peganum harmala*, a perennial plant thriving in arid ecosystems of Morocco [25]. Genome-based comparisons against the closest validly described relative, *A. marplatensis*, remained below species-level thresholds (ANI 91%; dDDH 38.8–38.9%), reinforcing the novelty of this species. This cross-host recovery is biologically coherent, as both host plants were sampled in comparable arid and semi-arid environments using similar isolation workflows. Overall, the data support a non-host-specific lineage shaped primarily by the environment rather than by association with *Citrullus colocynthis*.

In core-genome phylogenetic reconstructions, the three type strains formed well-supported, clearly separated lineages within the genus *Achromobacter*, each clustering closest to, but distinctly apart from, its nearest described species. This phylogenetic structure indicates that the novel taxa represent independent evolutionary lineages rather than recent variants of existing species, consistent with their genome relatedness indices.

The comparative circular genome maps revealed a pattern of broad genomic conservation together with localized regions of reduced nucleotide identity. This pattern is consistent with species-level divergence occurring primarily through variation in the accessory genome rather than disruption of the overall chromosomal backbone [42]. In this context, the divergent segments may correspond to flexible genomic regions, including loci shaped by horizontal gene transfer and mobile genetic elements, which are known to contribute to genome plasticity and lineage differentiation in bacteria. Thus, the genome maps complement the ANI, dDDH, and phylogenomic results by showing that the proposed novel species are not only separated by global genome-relatedness metrics but also by localized genomic differences relative to their closest type strains [43].

Phenotypic and genome-based characterization revealed patterns that may be consistent with adaptation to plant-associated niches. BioLog profiling showed limited carbohydrate utilization across all three type strains, with relatively higher use of organic acids and amino acids. This profile is consistent with the utilization of plant-derived substrates, which can shift toward non-sugar compounds under water-limited conditions [44]. However, phenotypic microarrays reflect physiological responses under defined assay conditions rather than absolute metabolic capacity [45]; therefore, these results should be interpreted cautiously and do not constitute evidence of restricted metabolism in natural environments.

Genome-based functional annotation provides complementary insights but remains predictive in nature. The three strains share a conserved genomic core, with some variation in annotated functions. Such patterns have been reported in plant-associated bacteria and may reflect differences in microenvironments within plant tissues [46], for example along gradients of oxygen, osmolarity, or nutrient availability [44]. Traits commonly associated with endophytic lifestyles, including motility, adhesion, and stress response, were identified through annotation [47]; however, their presence does not demonstrate activity or ecological function under in planta conditions. The recovery of a conspecific isolate from foliar tissue (AGC14) suggests that members of the *A. semiaridum* lineage could occur in multiple plant compartments, although this observation alone does not establish colonization dynamics or ecological roles [44].

Screening for plant-growth-related traits provides additional context but should be interpreted with caution. ACC deaminase activity was detected in all three type strains under the tested conditions, and this enzyme has been associated with modulation of plant ethylene levels under stress [48]. However, these observations are based on in vitro assays and do not demonstrate effects on plant performance [49]. Indole-3-acetic acid (IAA) production varied among strains, which is consistent with previous reports of condition-dependent expression of auxin-related traits [50]. No phosphate solubilization or siderophore production was detected under the assay conditions; this may reflect regulation of these traits rather than their absence, but this remains to be experimentally verified [51].

Metal tolerance assays indicated that all three strains tolerated elevated zinc concentrations, with variation in tolerance to copper and cadmium. These phenotypes are consistent with the physicochemical characteristics of the sampling environment, where intermittent exposure to metals and oxidative stress may occur. Nevertheless, these observations are based on laboratory conditions and do not directly demonstrate ecological function in situ.

Overall, the combined phenotypic and genomic data describe conserved and variable features among the strains that may be relevant to plant-associated lifestyles. However, the functional inferences presented here are derived from genome annotation and in vitro assays and do not constitute evidence of activity under natural or in planta conditions. Further experimental studies, including plant interaction assays and expression analyses, will be necessary to validate the ecological relevance and potential plant-associated functions of these strains.

## 5. Conclusions

This study describes three novel *Achromobacter* species associated with *Citrullus colocynthis* and *Peganum harmala* from arid and semi-arid environments, supported by a combination of genome-resolved taxonomy, comparative genomics, and phenotypic characterization. Core-genome phylogeny resolved the type strains (AGC39, AGC45, and AGC69) as distinct and well-supported lineages within the genus *Achromobacter*, and genome relatedness indices further supported their delineation at the species level. Based on these data, we propose *Achromobacter semiaridum* sp. nov. (AGC39, AGC93, AGC14), *Achromobacter colocynthi* sp. nov. (AGC45, AGC61, AGC86, AGC78), and *Achromobacter maghribensis* sp. nov. (AGC25, AGC27, AGC69).

Combined phenotypic and genome-based analyses identified a shared core set of traits, along with variation among lineages in substrate utilization profiles and tolerance to abiotic stress under the tested conditions. Functional annotation suggested the presence of genes putatively associated with metabolic processes and stress responses, although these inferences are based on genome annotation and do not demonstrate activity under natural or in planta conditions. Similarly, phenotypic assays indicated ACC deaminase activity across all three type strains, variable indole-3-acetic acid production, and tolerance to selected heavy metals; however, these observations were obtained under in vitro conditions and should not be interpreted as evidence of ecological function or plant-growth-promoting activity.

Comparative genome analyses revealed localized divergence relative to closely related type strains, providing additional support for species-level differentiation. Together, these results expand the current taxonomic framework of the genus *Achromobacter* and document its occurrence in plant-associated environments from semi-arid regions.

Further studies will be required to assess the ecological relevance of the observed traits and to determine whether these bacteria interact with plants under natural conditions. In particular, experimental validation through plant inoculation assays, gene expression analyses, and in situ investigations will be necessary to evaluate potential plant-associated functions and their significance in arid and semi-arid ecosystems.

## Figures and Tables

**Table 1:** Genome-based relatedness of *Achromobacter* strains to their closest type strains. Cc-R, *Citrullus colocynthis* roots; Cc-L, *Citrullus colocynthis* leaves; Ph-R, *Peganum harmala* roots; and Ph-L, *Peganum harmala* leaves.

Origin	Novel Strain	Closest Relative	ANI (%)	dDDH (%)
Cc-R	*Achromobacter semiaridium* AGC39	*A. piechaudii*	95.50	57
Cc-R	*Achromobacter semiaridium* AGC93	*A. piechaudii*	89.00	31
Cc-L	*Achromobacter semiaridium* AGC14	*A. piechaudii*	89.00	31
Cc-R	*Achromobacter colocynthi* AGC45	*A. spanius*	94.00	50
Cc-R	*Achromobacter colocynthi* AGC61	*A. spanius*	94.27	50
Cc-R	*Achromobacter colocynthi* AGC86	*A. spanius*	94.30	50
Cc-R	*Achromobacter colocynthi* AGC78	*A. spanius*	94.40	50
Cc-R	*Achromobacter maghribensis* AGC69	*A. marplatensis*	91.00	39
Ph-L	*Achromobacter maghribensis* AGC25	*A. marplatensis*	91.00	38.9
Ph-R	*Achromobacter maghribensis* AGC27	*A. marplatensis*	91.00	38.8

**Table 2:** Quantitative and qualitative screening of plant-growth-promoting traits in the novel *Achromobacter* AGC39, AGC45, and AGC69. Qualitative results are indicated as not detected (−), and quantitative values are provided where available. ΔA540 represents background-corrected absorbance at 540 nm, with higher values indicating a stronger ACC-associated colorimetric response.

PGPR Trait	AGC39	AGC45	AGC69	Method/Condition
Indole-3-acetic acid (IAA) production	~4.0 µg/mL	0 µg/mL	~10–11 µg/mL	Salkowski (+tryptophan); quantitative (standard curve) + qualitative colorimetric scoring
ACC deaminase activity	weak (ΔA540 = 0.035)	negative (ΔA540 = 0.020)	positive (ΔA540 = 0.080)	Honma & Shimomura colorimetric assay (α-ketobutyrate/DNPH)
Phosphate solubilization	(−)	(−)	(−)	Pikovskaya medium
Growth under iron chelation (DIP)	(−)	(−)	(−)	(CAS blue) + iron chelation growth (M9 + DIP)
Copper tolerance	≥50% relative growth up to ~5 mM	≥80% relative growth up to ~10 mM	≥80% relative growth up to ~10 mM	Metal-amended medium
Cadmium tolerance	≥50% relative growth up to ~5 mM	≥50% relative growth up to ~10 mM	≥50% relative growth up to ~10 mM	
Zinc tolerance	≥80% relative growth up to ~5 mM	≥80% relative growth up to ~5 mM	≥80% relative growth up to ~5 mM	
Hydrogen cyanide (HCN) production	(−)	(−)	(−)	Picrate qualitative assay

## Data Availability

Accession numbers for 16S rRNA gene sequences have been deposited in GenBank under the accession numbers PV706298, PV706320, PV706294, PV706299, PV706306, PV706317, PV706313, and PV706309. Genome sequence data for the bacterial isolates have been deposited in the Sequence Read Archive (SRA) at https://www.ncbi.nlm.nih.gov/sra/ (accessed on 9 July 2024), under the BioProject accession numbers: SRR29855769, SRR29855775, SRR29855796, SRR29855759, SRR29855772, SRR29855770, SRR29855771, and SRR29855774. The type strains of the ten novel bacterial species have been deposited in the Moroccan Coordinated Collections of Micro-organisms (CCMM) (https://www.ccmm.ma) (accessed on 29 January 2025), and their accession numbers are: CCMM B1338, CCMM B1340, and CCMM B1336. Genome assemblies are publicly available in Zenodo; the corresponding DOIs for each genome are provided in Table S3.

## Supplementary Materials

The following supporting information can be downloaded at: https://www.mdpi.com/article/10.3390/microorganisms14051019/s1. Table S1: Taxonomic identification of the novel *Achromobacter* isolates from *Citrullus colocynthis* and *Peganum harmala* based on 16S rRNA gene sequencing (Sanger method). Cc-L, *Citrullus colocynthis* leaves; Cc-R, *Citrullus colocynthis* roots; Ph-L, *Peganum harmala* leaves; and Ph-R, *Peganum harmala* roots. Table S2. Key features of the draft genome assemblies of the novel *Achromobacter* isolates recovered from *Citrullus colocynthis* and *Peganum harmala*. Table S3. Accession numbers and public repository links for the genome assemblies of the novel *Achromobacter* type strains. Figure S1. Biochemical profile of *Achromobacter colocynthi* AGC45 determined using the GEN III Biolog MicroPlate system. Figure S2. Biochemical profile of *Achromobacter maghribensis* AGC69 determined using the GEN III Biolog MicroPlate system. Figure S3. Biochemical profile of *Achromobacter semiaridium* AGC39 determined using the GEN III Biolog MicroPlate system. Figure S4. 16S rRNA gene phylogeny of *Achromobacter* isolates recovered from *Citrullus colocynthis*. The tree was inferred from partial 16S rRNA gene sequences (Sanger) following multiple-sequence alignment with MAFFT and maximum-likelihood reconstruction using FastTree (GTR + Gamma model). Tip labels correspond to isolate IDs. This analysis supports genus-level assignment to *Achromobacter* and is presented as a preliminary taxonomic screen; species-level delineation was resolved using whole-genome data.
