# Supplementary material for: Genome-Resolved Delineation of Three Novel Endophytic Achromobacter Species from Desert Medicinal Plants
Source: Microorganisms. 2026 Apr 30;14(5):1019. doi: 10.3390/microorganisms14051019 (PMC13209679; doi:10.3390/microorganisms14051019)
Supplement: Supplementary file 1 [file microorganisms-14-01019-s001.zip › microorganisms-4267447-supplementary.pdf]

Supplementary Information

Table S1. Taxonomic identification of the novel *Achromobacter* isolates from *Citrullus colocynthis* and *Peganum harmala* based on 16S rRNA gene sequencing (Sanger method). Cc-L, *Citrullus colocynthis* leaves; Cc-R, *Citrullus colocynthis* roots; Ph-L, *Peganum harmala* leaves; Ph-R, *Peganum harmala* roots.

Strain	Origin	Species	*length (bp)*	*Identity (%)*	*Coverage (%)*	*Accession number*
**AGC39**	Cc-R	*Achromobacter* sp.	817	100	100	PV706298
AGC93	Cc-R	*Achromobacter* sp.	470	96.86	100	PV706320
AGC14	Cc-L	*Achromobacter* sp.	795	98.77	99	PV706294
AGC45	Cc-R	*Achromobacter* sp.	809	97.12	100	PV706299
AGC61	Cc-R	*Achromobacter* sp.	827	99.67	100	PV706306
AGC86	Cc-R	*Achromobacter* sp.	730	97.51	100	PV706317
AGC78	Cc-R	*Achromobacter* sp.	744	97.90	100	PV706313
AGC69	Cc-R	*Achromobacter* sp.	816	99.59	100	PV706309
AGC25	Ph-L	*Achromobacter* sp.	832	99.8	100	*PV739401*
AGC27	Ph-R	*Achromobacter piechaudii*	798	98.4	94.33	*PV739374*

Table S2. Key features of the draft genome assemblies of the novel *Achromobacter* isolates recovered from *Citrullus colocynthis* and *Peganum harmala*.

Strain	Bacterial Species	Assembler	Largest Contig	Contigs	N50	Genome Length	GC (%)	Completeness (%)	Contamination (%)	Depth (X)	Protein coding sequence (CDS)	tRNA aa	16S rRNA (%)	Accession number (SRA)
**AGC39**	*A. semiaridium*	MaSuRCA	270,233	137	87,258	6,315,812	64.71	99.07	1.32	114	6,058	20	93.3	SRR29855769
AGC93	*A. semiaridium*	MaSuRCA	527,772	70	141,860	6,271,754	64.73	99.03	0,47	95	5,785	20	71,4	SRR29855775
AGC14	*A. semiaridium*	MaSuRCA	957,456	66	171,317	6,193,621	64.85	99.35	0.7	163	5,725	20	98.9	SRR29855796
**AGC45**	*A. colocynthi*	MaSuRCA	776,214	28	575,448	6,580,168	64.20	99.07	1.05	331	6,150	20	96.7	SRR29855759
AGC61	*A. colocynthi*	MaSuRCA	416,520	55	249,680	6,381,338	64.23	98.83	0.47	98	6,162	21	97	SRR29855772
AGC86	*A. colocynthi*	MaSuRCA	771,166	34	485,870	6,703,801	64,17	99.07	0.93	108	6,169	21	97	SRR29855770
AGC78	*A. colocynthi*	MaSuRCA	775,284	67	341,920	6,530,774	64.24	99.58	0.58	385	5,928	17	96,6	SRR29855771
**AGC69**	*M. maghribensis*	MaSuRCA	421,258	109	148,930	6,666,272	64.97	98.74	0.53	69	5,999	20	54.7	SRR29855774
**AGC25**	*M. maghribensis*	metaSPAdes	95,829	412	25,598	6,321,816	65,16	81.48	0	47	6,248	20	92	SRR29855733
**AGC27**	*M. maghribensis*	MaSuRCA	1,102,611	51	322,012	6,651,350	64.97	99.02	0.53	124	6,225	19	96.3	SRR29855732

Table S3. Accession numbers and public repository links for the genome assemblies of the novel *Achromobacter* type strains.

Strain	Zenodo DOI	Files deposited
AGC39	10.5281/zenodo.18530450	FASTA
AGC45	10.5281/zenodo.18530584	FASTA
AGC69	10.5281/zenodo.18530628	FASTA

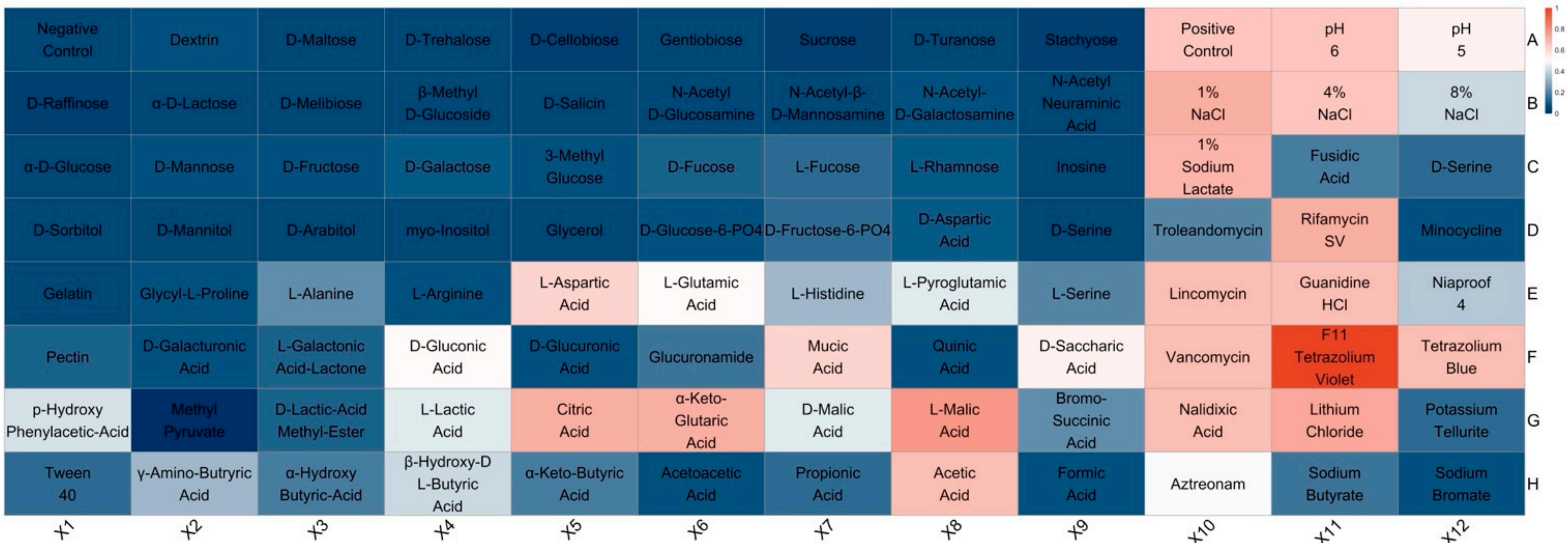

Figure S1. Biochemical profile of *Achromobacter colocynthi* AGC45 determined using the GEN III Biolog MicroPlate system.

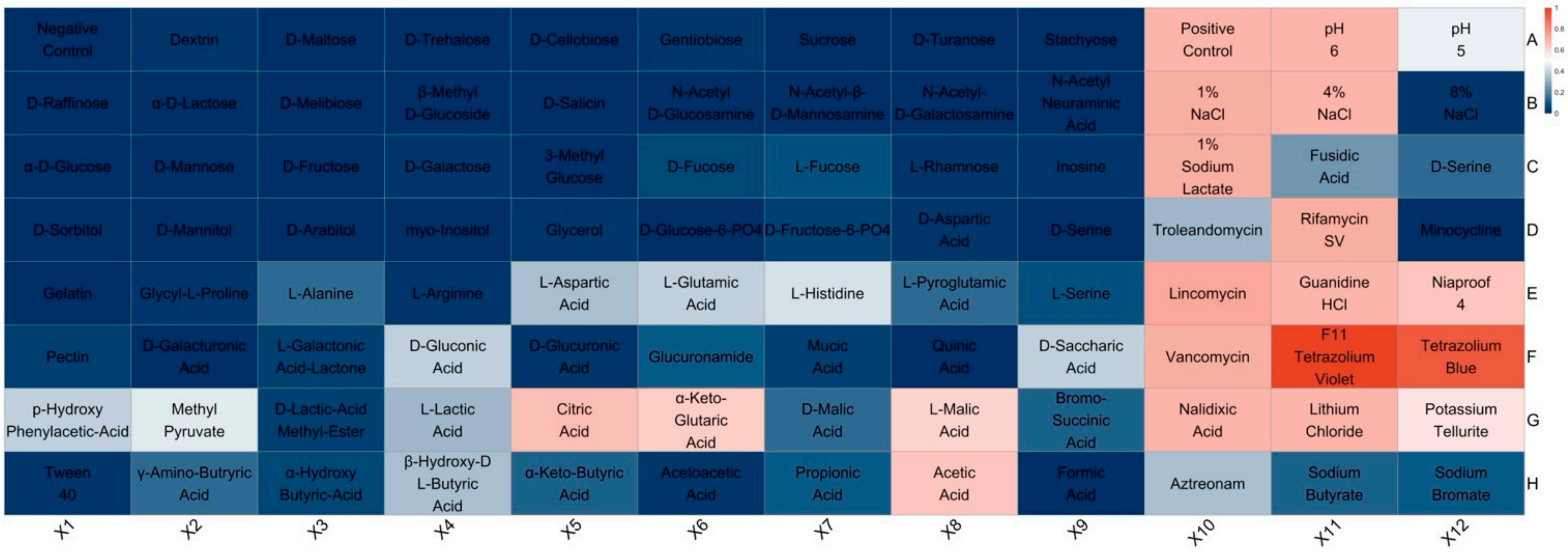

Figure S2. Biochemical profile of *Achromobacter maghribensis* AGC69 determined using the GEN III Biolog MicroPlate system.

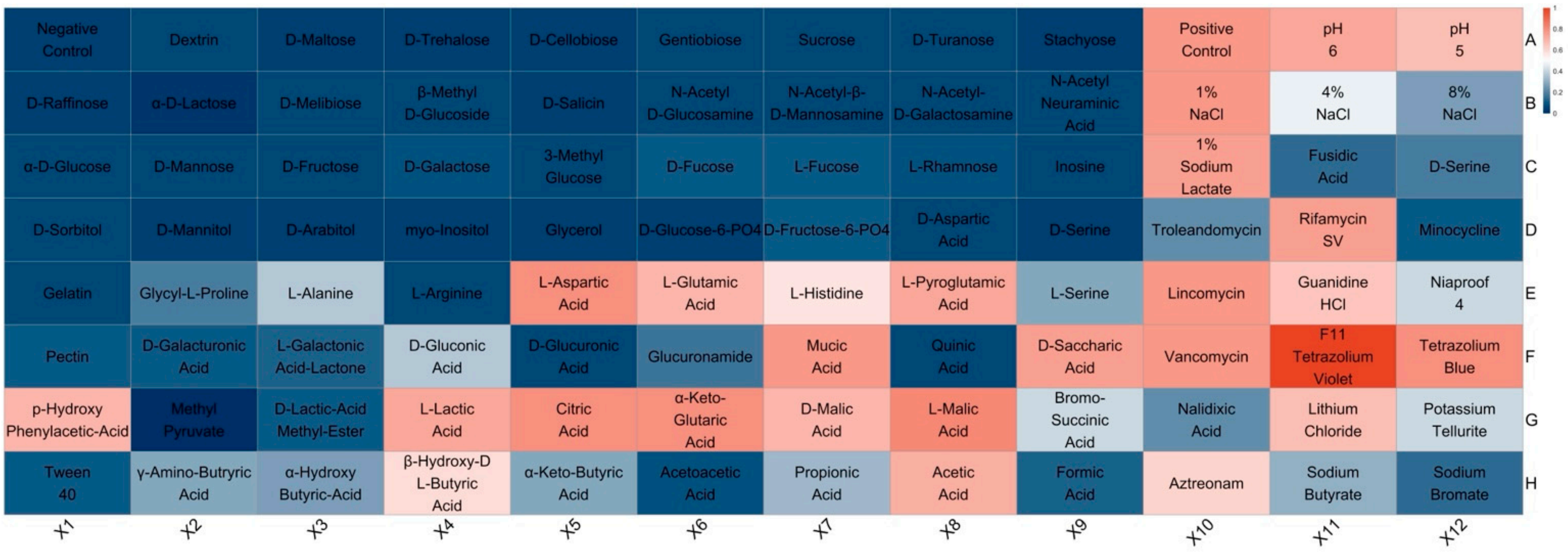

Figure S3. Biochemical profile of *Achromobacter semiaridium* AGC39 determined using the GEN III Biolog MicroPlate system.

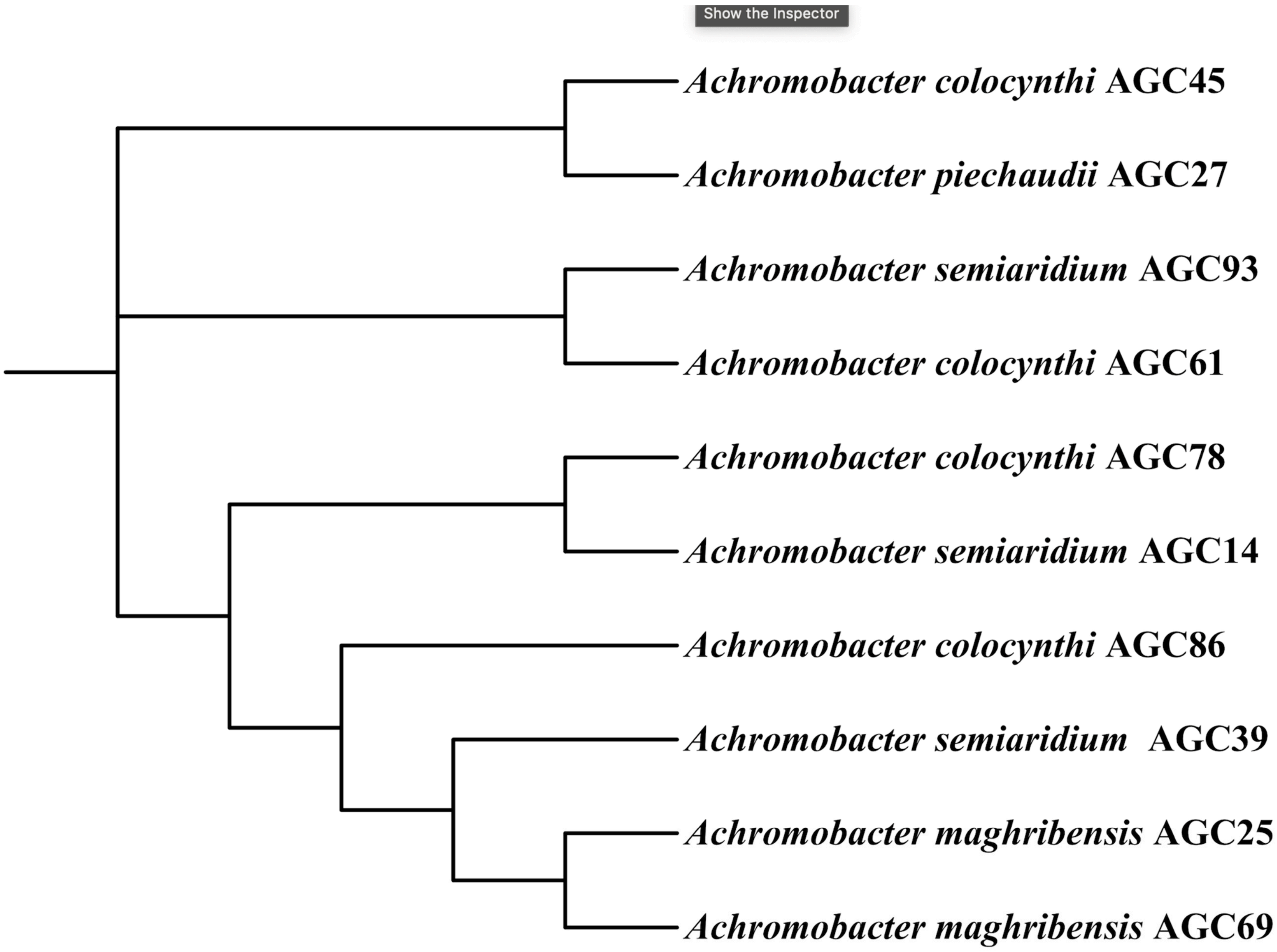

Figure S4. 16S rRNA gene phylogeny of *Achromobacter* isolates recovered from *Citrullus colocynthis*. The tree was inferred from partial 16S rRNA gene sequences (Sanger) following multiple-sequence alignment with MAFFT and maximum-likelihood reconstruction using FastTree (GTR+Gamma model). Tip labels correspond to isolate IDs. This analysis supports genus-level assignment to *Achromobacter* and is presented as a preliminary taxonomic screen; species-level delineation was resolved using whole-genome data.
